# Male–male behavioral interactions drive social-dominance-mediated differences in ejaculate traits

**DOI:** 10.1093/beheco/araa118

**Published:** 2020-11-28

**Authors:** Charel Reuland, Brett M Culbert, Erika Fernlund Isaksson, Ariel F Kahrl, Alessandro Devigili, John L Fitzpatrick

**Affiliations:** 1 Department of Zoology, Stockholm University, Svante Arrhenius väg 18B, Stockholm, Sweden; 2 Department of Integrative Biology, University of Guelph, Guelph, Canada

**Keywords:** agonistic interactions, condition, postcopulatory, precopulatory, sexual selection, sperm competition

## Abstract

Higher social status is expected to result in fitness benefits as it secures access to potential mates. In promiscuous species, male reproductive success is also determined by an individual’s ability to compete for fertilization after mating by producing high-quality ejaculates. However, the complex relationship between a male’s investment in social status and ejaculates remains unclear. Here, we examine how male social status influences ejaculate quality under a range of social contexts in the pygmy halfbeak *Dermogenys collettei*, a small, group-living, internally fertilizing freshwater fish. We show that male social status influences ejaculate traits, both in the presence and absence of females. Dominant males produced faster swimming and more viable sperm, two key determinants of ejaculate quality, but only under conditions with frequent male–male behavioral interactions. When male–male interactions were experimentally reduced through the addition of a refuge, differences in ejaculate traits of dominant and subordinate males disappeared. Furthermore, dominant males were in a better condition, growing faster, and possessing larger livers, highlighting a possible condition dependence of competitive traits. Contrary to expectations, female presence or absence did not affect sperm swimming speed or testes mass. Together, these results suggest a positive relationship between social status and ejaculate quality in halfbeaks and highlight that the strength of behavioral interactions between males is a key driver of social-status-dependent differences in ejaculate traits.

## Teaser

The winner takes it all? In the pygmy halfbeak fish, males who win fights are also likely to win fertilization bouts by producing superior ejaculates. Socially dominant males have faster swimming sperm and more viable ejaculates than subordinates, challenging the common conception that males need to specialize. However, dominant halfbeaks can only surpass subordinates in ejaculate quality if there is ample opportunity for fighting. If fighting between males is infrequent, no differences in ejaculates emerge.

## INTRODUCTION

Male–male competition can occur both before and after mating and is a powerful selective force that commonly generates variation in male reproductive success (Darwin 1871; Andersson 1994). Before mating (i.e., precopulatory), males in many species compete for access to females, with their social status often reflecting their relative success in fending off competitors and/or monopolizing access to females (Darwin 1871; Andersson 1994). Males can invest in a variety of traits to obtain a competitive advantage and attain a higher social status, such as the development of weapons (e.g., horns and antlers), larger body size, and changes in behavioral patterns, such as heightened aggression (Andersson 1994; Hardy and Briffa 2013). Male–male competition can continue after mating (i.e., postcopulatory) in polyandrous species, as the sperm of rival males compete for the fertilization of a female’s egg(s) (Parker 1970, 1998; Snook 2005; Pizzari and Parker 2009). Male reproductive success during postcopulatory sperm competition is typically determined by variation in aspects of their ejaculate, including variance in sperm number, viability, swimming speed, and morphology (Snook 2005; Pizzari and Parker 2009; Simmons and Fitzpatrick 2012; Fitzpatrick and Lüpold 2014). However, both precopulatory and postcopulatory competitive traits, which help attain a high social status and high ejaculate quality, are energetically costly to produce and maintain (Kotiaho 2001; Simmons et al. 2017). Thus, understanding how males balance their investment in precopulatory and postcopulatory competitive abilities represents a longstanding challenge in evolutionary biology that has important implications for our understanding of male reproductive strategies.

The relationship between male social status and ejaculate quality can vary depending on how social dominance influences male reproductive opportunities. Socially dominant males are expected to produce superior ejaculates if trait expenditure is condition dependent (Simmons and Kotiaho 2002; Schulte-Hostedde and Millar 2004; Schulte-Hostedde et al. 2005) and/or if social interactions lead to reduced capacity for subordinate individuals to invest in sexual traits (Lindsay et al. 1976; D’Amato 1988; Tilbrook et al. 2000). Indeed, positive covariance between precopulatory and postcopulatory competitiveness is observed in species where social encounters are frequent (Shen et al. 2015) or in highly social cooperatively breeding species, where persistent social interactions and constraints on independent breeding can lead to reproductive suppression of socially subordinate males (Fox et al. 1997; Koyama and Kamimura 1998, 2000; Faulkes and Bennett 2001; Hermes et al. 2005; Fitzpatrick et al. 2006). Furthermore, a positive relationship between precopulatory and postcopulatory competitiveness may occur when male condition predicts both social status and ejaculate expenditure. On the other hand, negative relationships between social status and ejaculates can occur when dominant males face trade-offs in allocating energy to precopulatory versus postcopulatory traits (Fitzpatrick et al. 2012; Parker et al. 2013; Lüpold et al. 2014; Simmons et al. 2017) or when social dominance hierarchies lead socially subordinate individuals to experience a higher risk of sperm competition, generating selection on subordinate males to invest more heavily in their postcopulatory competitiveness (Parker 1990; Parker et al. 2013). For example, experimentally induced developmental trade-offs lead to negative relationships between investment in precopulatory weapons and testes in some insect species (*Onthophagus* beetles, Simmons and Emlen 2006; *Narnia femorata*, Joseph et al. 2018; *Mictis profana*, Somjee et al. 2018), while there is ample evidence across a range of taxa that males mating in disfavored roles invest more in ejaculate quality (Montgomerie and Fitzpatrick 2009). Indeed, ejaculate quality often changes rapidly in response to experimental manipulation of social status (*Salvelinus alpinus*, Rudolfsen et al. 2006; *Gallus gallus*, Cornwallis and Birkhead 2007; Pizzari et al. 2007). Thus, both positive and negative phenotypic correlations between precopulatory and postcopulatory traits can emerge, depending on how social status influences male reproductive opportunities. Adequately understanding the link between social status and ejaculate traits, therefore, requires understanding how social interactions influence male reproductive opportunities in an experimental framework that allows causality to be investigated.

The social environment can also play a key role in determining the relationship between social status and ejaculates. Male sexual strategies can be influenced by the presence and availability of females (Warner et al. 1995; Sæther et al. 2001; Cornwallis and Birkhead 2007). For example, Procter et al. (2012) demonstrated that the strength and shape of multivariate selection gradients on male traits used during male–male competition were dependent on the presence or absence of females in a leaf-footed cactus bug, *Narnia femorata*. Moreover, exposure to females can stimulate male sex hormone production (*Carassius carassius*, Olsén et al. 2006; *Ozotoceros bezoarticus*, Villagrán and Ungerfeld 2013), increase sperm production (*Oncorhynchus* mykiss, Olsén and Liley 1993; *Poecilia reticulata*, Bozynski and Liley 2003), and lead to increased sperm swimming speed (*Poecilia reticulata*, Gasparini et al. 2009). Yet, while numerous studies have assessed plasticity in ejaculate traits in response to varying male socio-sexual environments (Kelly and Jennions 2011), the relationship between social status and ejaculates has rarely been considered in social environments that varied male access to females (Cornwallis and Birkhead 2007). Because social status can influence a male’s access to females, and access to females can influence how selection acts on male precopulatory and postcopulatory traits, gaining a robust understanding of the relationship between social status and ejaculate quality requires deconstructing this complex network of social interactions.

In this study, we investigated the relationship between male social status and ejaculate traits in the pygmy halfbeak *Dermogenys collettei*, a small, internally fertilizing fish native to freshwater environments in south-east Asia (Meisner, 2001; Greven, 2010). Halfbeaks are loosely shoaling fish, living in mixed-sex groups where frequent intrasexual and intersexual interactions create ample opportunity for sexual selection to act (Greven 2010; Ho et al. 2015). Halfbeaks perform frequent agonistic interactions between males, including aggressive displays, displacements, and overt competition, when males interlock their elongated jaws (called beaks) to “wrestle” until a winner is determined (Berten and Greven 1991; Greven 2006). Male–male competition continues after mating, as females mate multiple times during the same reproductive episode and can store sperm from previous matings (Reuland C, personal observation). Consequently, ejaculate traits in male halfbeaks are expected to be under strong selection owing to a high risk of sperm competition. The expected strong selection on male halfbeaks to compete during both precopulatory and postcopulatory bouts makes this species an excellent candidate to study the relationship between social and sperm competition in tandem. In this study, we first investigated the relationship between male social status and sperm swimming speed and how male access to females may influence this relationship. We then experimentally manipulated the intensity of male–male interactions to determine if agonistic interactions between dominant and subordinate males mediate social-status-dependent differences in ejaculate traits.

## Material and Methods

### Study populations and rearing conditions

Two experiments were performed on sexually mature halfbeaks that were F1 or F2 descendants of fish originating from two different sources. Experiment 1 used fish descendant from adults obtained by a commercial supplier (Ruinemans Aquarium B.V., Montfoort, The Netherlands), while Experiment 2 used descendants of a wild-caught parental population from the Tebrau River, Malaysia. For both experiments, juvenile fish were sexed at the initial period of sexual development at around 2 months of age. Male halfbeaks were then housed in same-sex tanks at a range of densities (approx. 15–30 males per tank) until sexual maturity (~4 months). Differences in housing densities during development can influence allocation to reproductive traits (Gage 1995). However, as densities were not biased in one treatment or experiment, any potential effects of rearing density are more likely to add statistical noise than to systematically influence the direction of observed effects. At sexual maturity, males were transferred to individual tanks housing a female to allow males to gain mating experiences before entering the experiment. After a week, the female was removed and males were kept in isolated tanks for another 3 weeks, at which point males entered the experiments. Mating and isolating males prior to the treatment ensured that sperm reserves were replenished. All tanks were oxygenated and contained ~2 cm of gravel and live and artificial plants. Fish were maintained on a 12:12 light:dark cycle at 27 °C and fed daily with a mix of ground flake flood, freeze dried *Artemia,* and once per week additional *Drosophila melanogaster.* Experiments were approved by the Stockholm Animal Research Ethical Board (permit number 3867-2020 and 2393-2018).

### Experiment 1: social status, socio-sexual environments, and investment in reproductive and condition traits

The influence of social status on male behavior, physiology, and reproductive traits was assessed under a range of socio-sexual environments. Males were photographed 1 day prior to the start of the experiment using a photo chamber (30 × 20 × 20 cm) fitted with a scale. Using these images, body length (from the tip of the lower beak to the caudal peduncle; see Reuland et al. 2019) was measured and size-matched pairs of males, henceforth called dyads, were created. Males in each dyad were statistically indistinguishable in total body length (average absolute difference within dyad ± standard error [SE]: 0.44 ± 0.10 mm, *n* = 32; paired two-sample *t*-test assuming equal variance: *t*(31) = 0.30, *P* = 0.77). Male dyads were placed in experimental tanks (40 × 25 × 30 cm; Supplementary Figure S1) and allowed to freely interact and establish social dominance hierarchies. Experimental tanks were divided into two chambers, with male dyads being placed in the larger chamber (26 × 25 × 30 cm). A removable opaque divider separated the larger and smaller chamber. Since we were interested in assessing the influence of social status across different socio-sexual contexts, male dyads were allocated to one of three experimental treatments that varied male access to females, including 1) a “no female” treatment (*n* = 9), where the smaller chamber was left empty, 2) a “visual access to female” treatment (*n* = 12), where the female was placed in the smaller chamber and a transparent divider was added (in addition to the opaque barrier) that separated the female from the male dyad, and 3) a “free access to female” treatment (*n* = 11), where females could freely interact with the male dyad, allowing both visual and physical contact among all fish (Supplementary Figure S1).

Male dyads were observed two times per day for 20 min on Days 1, 2, 6, and 10 to determine social status. Observational times were chosen to both capture frequent agonistic interactions early on during the establishment of social roles (Days 1 and 2), as well as to observe the stability of the formed dominance hierarchies (Days 6 and 10). During the initial behavioral observation on the morning of Day 1, the opaque partition was lifted to give males visual access to a female (visual access to female treatment) or physical access to a female (free access to female treatment). To standardize handling, partitions to the empty chamber were also lifted in the no-female treatment. Behaviors were recorded by applying a continuous sampling method and recording all the activity that occurred while the animals were observed (all activities within the dyad). Male social status within each dyad was determined by recording displacement behaviors, where one (aggressor) male approaches another or exerts an agonistic behavior and, as a consequence, the opposing male increases the distance between the two males to more than two-thirds of a body length again (Supplementary Table S1). Dominance indexes were calculated for both males in the dyads using the formula: (1 − $\frac{Displacements\;by\;rival\;male}{Total\;number\;of\;displacements\;in\;the\;dyad}$), where values of 1 indicate complete social dominance (males only displaced rival males) and values of 0 indicate complete social subordination (males were only displaced by rival males). We considered social dominance to be clearly resolved when one male in the dyad displaced the rival male in >70% of the interactions, which was the case in all dyads (average ± SE dominance index for males classified as dominant: 0.98 ± 0.01, *n* = 32, range: 0.75–1). Furthermore, to assess the frequency of male–male agonistic interactions within dyads, total agonistic behaviors between males (including gill flare, parallel swimming, chasing, beak-locking, and biting; see Supplementary Table S1) were recorded.

On the morning of Day 11, males were transferred to isolated tanks and given new anonymous IDs in preparation for data collection (i.e., males were processed blind to their social status or socio-sexual treatment). Males were photographed in the photo chamber and then euthanized in a benzocaine solution (final concentration 400 mg benzocaine in 1 L diH_2_O, where initial stock solution = 100 mg benzocaine to 1 mL Ethanol). After euthanasia, males were rinsed in distilled water and then placed on their side onto a slide covered with saline solution (0.9 % NaCl in diH_2_O) and viewed under a dissecting scope (S9 stereo microscope, Leica Microsystems, Wetzlar, Germany). Because male halfbeaks are partially transparent, the posterior part of the testicular duct is visible externally (Downing and Burns 1995). The testicular duct transfers sperm to the andropodium, a modified anal fin used to transfer sperm to females (Meisner and Burns 1997). Sperm were extracted into a saline solution by applying gentle pressure with a blunt instrument to the posterior part of the testicular duct. Halfbeak sperm cells are arranged in unencapsulated bundles called spermatozeugmata, with sperm tails on the outside and sperm heads facing toward the inside of the aggregate (Downing and Burns 1995). Spermatozeugmata structure remains largely intact when sperm are stripped into a saline solution and cells remain largely inactivated, assuring that sperm bundles can be collected before activation. The saline/sperm solution was then transferred into an equal volume of Hanks’ balanced salt solution (modified, with sodium bicarbonate, without phenol red, H8264, Sigma-Aldrich, St. Louis, MO) and briefly mixed to activate the sperm. After activation, sperm were transferred to a polyvinyl alcohol-coated multiwell slide (Multitest slide, MP Biomedicals, Santa Ana, CA) and sperm swimming speed (median curvilinear track velocity VCL, average path velocity VAP, and straight line velocity VSL) was analyzed using a computer-assisted-sperm-analysis system consisting of a computer and camera attached to a microscope (ISAS V1 system, PROiSER R+D, Paterna, Spain). As VCL was strongly positively correlated with both VAP (*r* = 0.92, *n* = 63) and VSL (*r* = 0.97, *n* = 63), and since cell trajectories were not expected to be linear due to the lack of any egg attraction or ovarian fluid effects in this study, we used VCL in all subsequent analyses (note that results are consistent irrespective of the metric used).

We also recorded the change in body area during the experiment, as well as male condition (see below) and liver mass after the experiment. The change in body area (omitting the fins) during the experiment was measured as an approximation for a male’s growth using the photos taken 1 day before dyad formation and on the 11th day of the experimental treatment. We analyzed body growth using photographs. Photographs were used rather than weighting fish before the experiment to minimize the handling of the animals before the formation of male dyads. To assess male condition, after males were euthanized at the end of the experiment, they were lightly dried with paper towel, measured for body length (the distance from the eye to the caudal peduncle excluding the beak), and weighed to the nearest 0.01 mg on a fine scale (XS105, Mettler Toledo, OH). Condition factor was calculated using the formula: (body mass/body length^3^) × 100, which is commonly used as an indicator of overall fish health (Ricker 1975; Froese 2006). In teleost fishes, the liver is an important store for energy reserves and relative liver mass is commonly used as an indicator of a fish’s energy status (Tyler and Dunn 1976; Wootton et al. 1978; Culbert and Gilmour 2016). Therefore, after weighting the fish, males were dissected and their liver weighed. Together, these metrics are likely candidate traits to assess energy reserves in halfbeak fishes, as they are commonly used in other fish species.

Males investing more in postcopulatory competitive traits typically have larger testes relative to their body mass (Simmons and Fitzpatrick 2012). Thus, as an indicator for male expenditure on postcopulatory traits, testes of males were dissected and weighted.

### Experiment 2: social status, male–male interactions, and ejaculate traits

We investigated how the experimental manipulation of male–male interactions influences social-status-dependent differences in ejaculate traits. This experiment focused in detail on responses in a range of ejaculate traits, as Experiment 1 suggested that ejaculates are responsive to differences in social status among males (see Results). Males were photographed up to 3 days prior to the formation of male dyads (see Experiment 1). Size-matched male dyads (average absolute difference within dyad ± SE: 0.38 ± 0.09 mm, *n* = 44; paired two-sample *t*-test assuming equal variance: *t*(43) = −0.98, *P* = 0.33) were placed in experimental tanks that were identical to the “visual access to female” treatment in Experiment 1. We manipulated the extent to which males in a dyad were able to interact. Dyads were placed in tanks where 1) males could interact freely (as in Experiment 1), henceforth called “− Refuge” treatment (*n* = 24), or 2) the opportunity for males to interactions was experimentally reduced by introducing a refuge, henceforth called “+ Refuge” treatment (*n* = 20). Experimental reduction in male interactions was achieved through the addition of a small opaque wall (5 cm width) placed in the chamber, which created a refuge, reduced visual contact between the males, and thus reduced the frequency of male–male interactions (see Results).

Male dyads were observed for 20 min on three to four occasions (as in Experiment 1) and social status was determined based on behavioral interactions between the males (as in Experiment 1). Dominance index scores for the dominant males in each dyad ranged from 0.54 to 1 (average ± SE: 0.87 ± 0.02, *n* = 44). Eight dyads (four in the “− Refuge” and four in the “+ Refuge” treatments) were omitted from the final analysis as, in each dyad, one male did not win at least 70% of agonistic encounters and thus the dominance hierarchy was unclear (scores between 0.54 and 0.7). This reduced the final sample size for analysis to *n* = 20 for the “− Refuge” treatment and *n* = 16 for the “+ Refuge” treatment. We present a statistical analysis including these dyads in Supplementary Table S2. Comparisons of frequencies of agonistic interactions between treatments only incorporated behavioral data collected on days common to all dyads, that is, Days 1 and 8. Frequencies of male–male agonistic interactions within dyads were recorded as described for Experiment 1 (i.e., gill flare, parallel swimming, chasing, beak-locking, and biting; see Supplementary Table S1).

On the morning of Day 13, males were transferred to isolated tanks and given new anonymous IDs (to ensure that experimenters were blind to male social status and treatment). Males were lightly sedated (final concentration 67 mg benzocaine in 1 L diH_2_O, where initial stock solution = 100 mg benzocaine to 1 mL ethanol), stripped of sperm, and sperm swimming speed was measured as described in Experiment 1. As for Experiment 1, we present only VCL in all subsequent analyses, but results were consistent irrespective of the metric used for sperm swimming speed. A subset of the sperm was dyed with a solution of propidium iodide (ex503-530/em640) and acridine orange (ex536/em600-640) in order to assess sperm viability (Vitaltest, NordicCell, Copenhagen, Denmark). Propidium iodine and acridine orange are fluorogenic compounds binding to nucleic acids and thus staining sperm nuclei. The cell-permeable acridine orange stains both alive and dead cells green, while propidium iodine can only enter cells with a loss of membrane integrity, staining them red. Thus, alive cells are stained green, while dead cells are stained red. In instances where cells were dyed both green and red, meaning that both dyes had entered the cell, cells were classified as dead. The light-sensitive dyes were kept in the dark at all times, as were sperm cells after the addition of the dye. After adding the dyes, sperm was briefly mixed and then transferred to a microscopic slide. Cells settled on the slide for about 2 min (average ± SE: 130 ± 2.27 s, *n* = 65) in the dark. After all cells had settled on one focal plane, images of the cells were taken under a microscope (×200 magnification; UB 200i Series Microscope and C13-ON camera, PROiSER R+D, Paterna, Spain). Viability was assessed by counting the proportions between red or green-red (dead) and green (alive) cells. Samples where less than 100 cells were recorded were omitted from the final analysis to ensure the reliability of the data (*n* = 9). From nonfixed samples, images of sperm cells were taken (×400 magnification; UB 200i Series Microscope and C13-ON camera, PROiSER R+D, Paterna, Spain) and 20 morphologically normal (i.e., with all sperm components clearly distinguishable) sperm per male were measured to assess sperm morphology differences. Sperm morphology was assessed by measuring the length of the 1) sperm head, 2) midpiece, 3) flagellum using ImageJ v1.52r (Schneider et al. 2012), and 4) total sperm length was assessed by summing respective head, midpiece, and flagellum measurements. Lastly, sperm samples were fixed by adding 5% formalin, and sperm cells were counted using a microscope and a counting chamber (Neubauer Improved). Samples with low final sperm counts (<200) were removed from the final statistical analysis (*n* = 2).

### Statistical analysis

#### Experiment 1

The effect of male access to females on male agonistic behavior was analyzed in a linear model with the average frequency of agonistic interactions between male dyads per 20 min (per observational unit) as the response variable and experimental treatment as the independent variable. Agonistic interactions were square-root transformed to normalize the data and ensure a better fit of the model.

To determine the effects of male social status and male access to females on sperm swimming speed, testes mass, male condition factor, and change in body area during the experiment, we constructed linear mixed-effects models with the respective trait as the response variable. Male social status and treatment (male access to females), as well as their interaction term, were treated as independent variables. Dyad ID was included in the model as a random effect. To prevent overfitting, liver mass was analyzed irrespective of dyad ID as a linear model with male social status, treatment, and their interaction term as independent variables. When analyzing sperm swimming speed, data were weighted by the number of motile sperm measured for each male to account for variation in measurement number among males (number of motile sperm cells average ± SE: 133 ± 9, *n* = 63, range 25–315). For testes and liver mass, body mass was included as a covariate in the model to account for allometric effects.

Additionally, we tested the relationship between sperm swimming speed and male body condition by constructing a linear model using within-dyad differences in sperm swimming speeds (dominant—subordinate male) as the dependent variable and differences in body condition, access to females, and their interaction term as covariates.

#### Experiment 2

The effect of refuge provision on male agonistic behavior was analyzed in a linear model with the average frequency of agonistic interactions between male dyads per 20 min (per observational unit) as the response variable and experimental treatment (“−” and “+” refuge) as the independent variable. Agonistic interactions were square-root transformed to normalize the data and ensure a better fit of the model.

Experimental treatments (“−” and “+” refuge) were performed sequentially (first “− Refuge” dyads and then dyads of “+ Refuge” treatments) and not simultaneously as in Experiment 1. To account for the sequential nature of the treatments, statistical analyses were performed using standardized trait values, where absolute sperm trait values were divided by treatment means (individual value/mean value within the respective refuge, i.e., “−” or “+,” treatment group). Comparing standardized values accounts for potential block effects that can emerge from sequential sampling and thus allows for more appropriate comparisons of treatment effects. Linear mixed-effects models were constructed with either average sperm swimming speed, sperm count, or sperm head length as the dependent variable; male social status, treatment (“−” and “+” refuge), and their interaction effect as independent variable; and dyad ID as a random effect. To prevent overfitting, sperm viability, sperm midpiece length, sperm tail length, and total sperm length were analyzed irrespective of dyad ID as linear models with male social status, treatment, and their interaction term as independent variables. However, results were consistent irrespective of the inclusion or exclusion of dyad ID. As in Experiment 1, sperm swimming speed data was weighted by the number of motile cells recorded to account for variation in the number of sperm measured among males (number of motile sperm cells average ± SE: 320 ± 30, *n* = 63, range 21–989). Sperm count was square-root transformed to fit the assumptions of normality. Likewise, midpiece length was transformed using a standardized Yeo–Johnson transformation (λ = 5, mean = 6.31, standard deviation = 1.33; Yeo and Johnson 2000) included in the VGAM package (Yee 2010).

All analyses were completed using R version 4.0.0 (R Core Team 2020). Significant effects were obtained using the “Anova” function included in the “car” package (Fox and Weisberg 2011). Nonsignificant interaction effects were dropped from final models. Model fit was assessed through visual inspection of the residuals, with outliers being dropped from the final model (i.e., one measurement for frequencies of agonistic interactions in Experiment 1 and one measurement for sperm viability in Experiment 2; see Results and Supplementary Table S3).

## RESULTS

### Experiment 1: social status, socio-sexual environment, and investment in reproductive and condition traits

**Figure 1:**
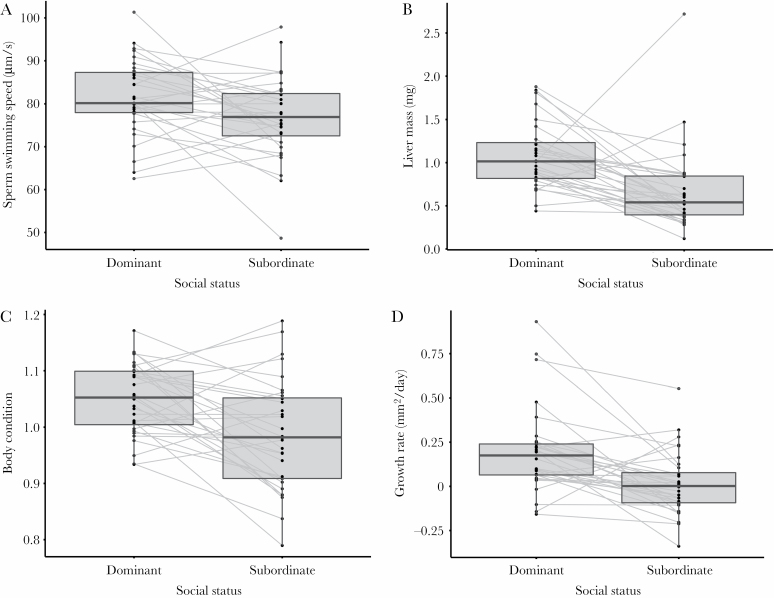
The effect of social status on investment in reproductive and condition traits. Individual values are presented for dominant and subordinate males for (a) sperm swimming speed, (b) liver mass, (c) body condition at the end of the experiment, and (d) growth rates during the experiment. Points represent individual values, and males within the same dyad are linked by a gray line. Lines are absent in cases where values for only one male within a dyad were present. Box plots summarize the 10th, 25th, 50th (median), 75th, and 90th percentiles among males classified as being socially dominant or subordinate. Note that, although raw liver mass is reported for size-matched dyads, statistical analyses accounted for male body mass. Full model outputs are presented in Table 1a. A visual representation of the effect of male access to females can be found in Supplementary Figure S3.

Dominant and subordinate males differed in reproductive and condition-related traits. After 10 days of social interactions, dominant males produced faster swimming sperm than subordinate males (Figure 1; Table 1a). Dominant males grew faster during the experiment, had larger livers (correcting for body size), and were in better condition than subordinates at the end of the experiment (Figure 1; Table 1a). Differences in sperm swimming speeds of dominant and subordinate males within dyads were not influenced by differences in body condition between males (χ ^2^_(1,31)_ = 1.35, *P* = 0.26) or access to females (χ ^2^_(2,31)_ = 0.27, *P* = 0.76). Furthermore, no differences in testes mass were recorded between males of contrasting social status (Figure 1; Table 1a).

**Table 1:** The effect of social status on male reproductive and condition traits. (a) Experiment 1 examined the effects of male social dominance status and socio-sexual environment (i.e., access to females) on male investment in reproductive (sperm swimming speed and testes mass) and condition (liver mass, body condition, and growth rate) traits. Experiment 2 (b) examines the effect of male social dominance status and male–male interactions, which were experimentally manipulated through the addition of a refuge (i.e., “− Refuge” treatment, without a refuge, and “+ Refuge” treatment, with an added refuge) on ejaculate traits (sperm swimming speed, viability, number and sperm head, midpiece, flagellum, and total length). The number of experimental dyads, and the total sample size (*n*) of males are presented for each model. Sample sizes (*n*) differ from the total number of dyads in cases where data could not be collected from males due to technical issues. Models assessing testes and liver mass included male body mass as a covariate to account for allometric effects. Nonsignificant interaction terms were dropped from all final models. Significant effects are presented in bold text

Response variable	Dyads	*n*	Predictors	χ ^2^	*P*
(a) Experiment 1					
Sperm swimming speed	32	63	Dominance status	8.84	**<0.01**
			Access to females	0.69	0.71
Testes mass	32	64	Dominance status	1.94	0.16
			Access to females	0.94	0.63
			Body mass	10.51	**<0.01**
			Dominance status × Body mass	7.74	**<0.01**
Liver mass	32	64	Dominance status	12.90	**<0.001**
			Access to females	2.98	0.23
			Body mass	17.61	**<0.001**
Body condition	32	64	Dominance status	12.79	**<0.001**
			Access to females	1.71	0.43
Growth rate	32	64	Dominance status	17.41	**<0.001**
			Access to females	2.82	0.24
(b) Experiment 2					
Sperm swimming speed	34	63	Dominance status	1.06	0.30
			Refuge	0.04	0.83
			Dominance status × Refuge	10.67	**<0.01**
Sperm viability^a^	32	58	Dominance status	0.61	0.44
			Refuge	0.004	0.95
			Dominance status × Refuge	4.91	**0.031**
Sperm count	36	65	Dominance status	0.26	0.61
			Refuge	0.68	0.41
Sperm head length	35	64	Dominance status	0.66	0.42
			Refuge	0.08	0.78
Sperm midpiece length	35	64	Dominance status	0.97	0.33
			Refuge	0.06	0.82
Sperm tail length	35	64	Dominance status	0.83	0.36
			Refuge	0.00	1.00
Total sperm length	35	64	Dominance status	0.31	0.58
			Refuge	0.00	1.00

^a^The interaction term for sperm viability was significant (χ ^2^_(1,57)_ = 6.21, *P* = 0.016) when data from one outlier male with low sperm viability (0.44 %) was removed from the analysis.

Male access to females had little influence on reproductive or condition-related traits, having no effect on sperm swimming speed, male condition, and body growth rate, nor liver and testes mass (Table 1a). However, access to females influenced agonistic interactions between males, with more frequent agonistic interactions observed when males had neither visual nor physical contact with females (*F*_(2,29)_ = 2.41, *P* = 0.11, the effect was significant *F*_(2,28)_ = 5.82, *P* < 0.01 when data from one dyad with high level of agonistic interactions, about 2.2× higher than the average, was removed from the analysis) (Supplementary Figure S2).

### Experiment 2: social status, male–male interactions, and ejaculate traits

**Figure 2:**
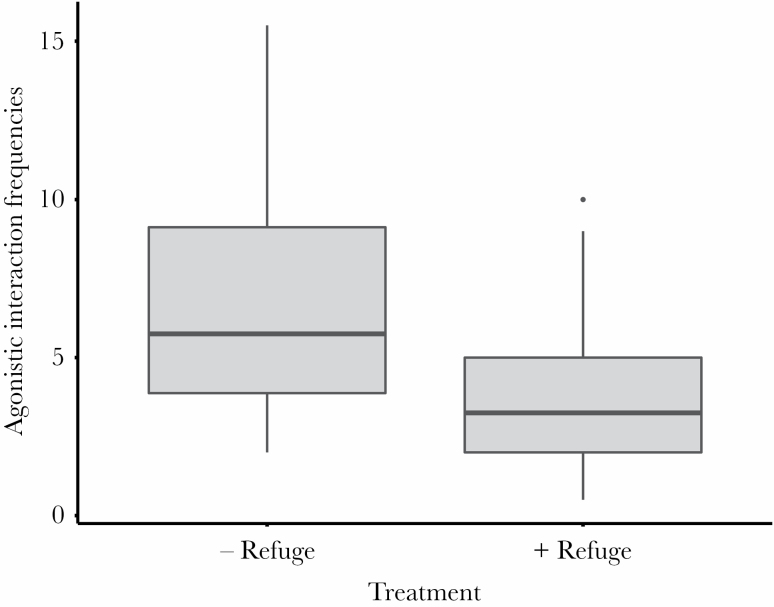
Differences in male–male agonistic interaction frequencies between refuge treatments that experimentally manipulated the opportunity for male–male interactions. The average frequencies of agonistic interactions per 20-min observation is presented for dyads in the “− Refuge” and the “+ Refuge” treatment. Box plots show the 10th, 25th, 50th (median), 75^th^, and 90th percentiles of agonistic interactions between males within the dyads in each refuge treatment.

**Figure 3:**
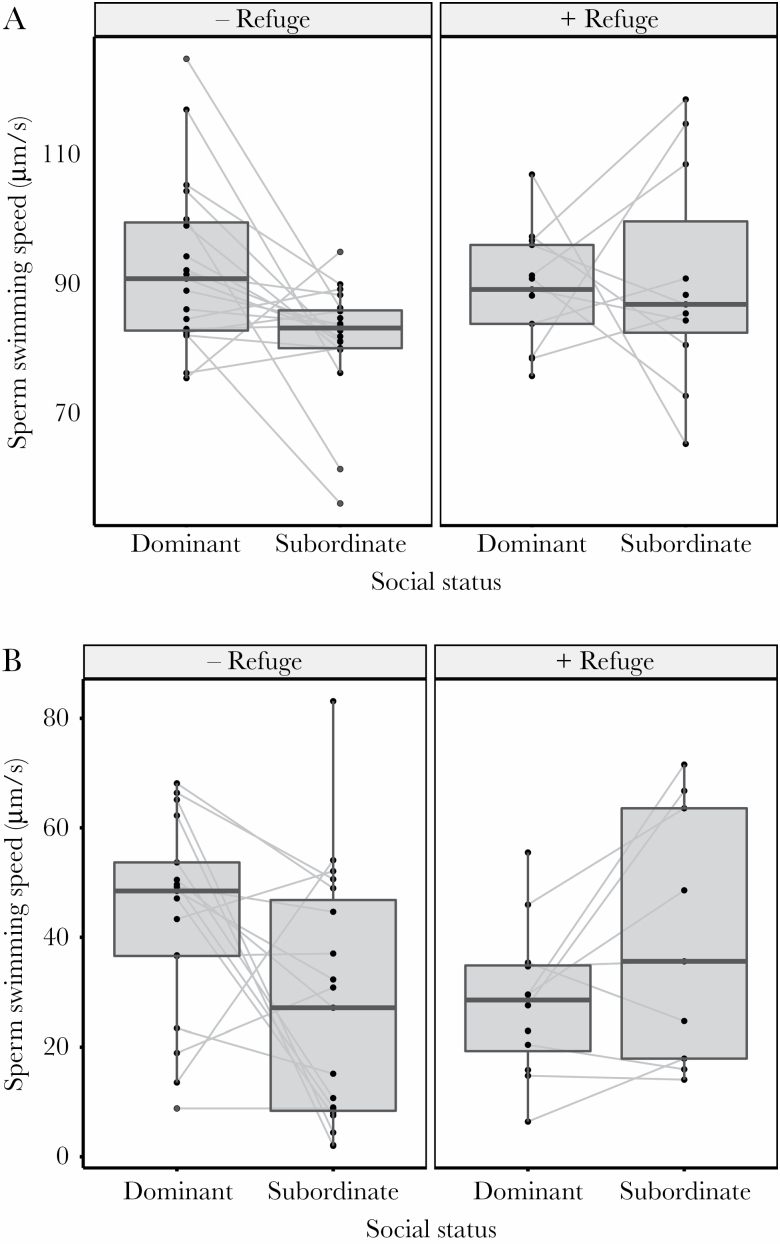
The effect of social status and male–male interactions on (a) sperm swimming speed and (b) viability. Individual values are presented for dominant and subordinate males in the “− Refuge” and the “+ Refuge” treatment, where agonistic interactions were experimentally reduced. Points represent individual values, and males within the same dyad are linked by a gray line. Lines are absent in cases where values for only one male within a dyad were present. Box plots summarize the 10th, 25th, 50th (median), 75^th^, and 90th percentiles among males classified as being socially dominant or subordinate. For sperm viability, data from one outlier male with low sperm viability (0.44 %) was removed from the representation. Full model outputs are presented in Table 1b.

Agonistic interactions between males were significantly less frequent (42.8 % reduction) in tanks with the addition of a small partition (i.e., the “+ Refuge” treatment) compared to when males could interact freely (i.e., the “− Refuge” treatment; *t*(34) = −2.58, *P* = 0.014; Figure 2). This experimentally induced reduction in male–male behavioral interactions influenced social-dominance-mediated differences in ejaculate traits. After 13 days of social interactions, dominant males had faster-swimming sperm compared to subordinates, but only when males interacted in experimental tanks with no additional refuge beyond floating plants (Figure 3; Table 1b). Such social-dominance-mediated differences in ejaculate traits are consistent with the results reported in Experiment 1 (above). In the “+ Refuge” treatment, where male–male agonistic interaction frequencies were experimentally reduced, sperm swimming speed did not differ between dominant and subordinate males (Figure 3; Table 1b). Sperm viability was also higher in dominant males compared to subordinate males in the “– Refuge” treatment, but no differences in sperm viability between dominant and subordinate males were detected in the “+ Refuge” treatment (Figure 2; Table 1b). Neither male social status nor presence or absence of a refuge influence sperm count or sperm morphology (sperm head, midpiece, flagellum, and total length; Figure 2; Table 1b).

## DISCUSSION

We demonstrate that social status mediates differences in ejaculate traits in pygmy halfbeaks of two distinct populations and that these effects are dependent on the frequency of male–male agonistic interactions. Males that were dominant in experimentally constructed dyads produced faster swimming sperm than subordinate males. By experimentally manipulating the opportunity for male–male behavioral interactions through the introduction of a refuge, we found that social-status-mediated differences in sperm swimming speed were dependent on the frequency of agonistic interactions between males. Moreover, when examining ejaculate traits in greater detail, we found that sperm viability was also higher in dominant compared to subordinate males, but this difference was also dependent on the frequency of male–male agonistic interactions with differences only being observed when agonistic interactions were frequent. Together, these results suggest a positive relationship between precopulatory and postcopulatory competitive abilities (social status and ejaculate quality) in pygmy halfbeaks and that differences in ejaculates quality arise due to direct agonistic interactions between males. Surprisingly, the manipulation of male access to females revealed that female interactions had little effect on male’s investment in traits.

Social-status-mediated differences in ejaculate traits can have far-reaching fitness consequences. Sperm swimming speeds predict fertilization success in both competitive (Birkhead et al. 1999; Donoghue 1999; Gage et al. 2004; Denk et al. 2005) and noncompetitive scenarios (Donnelly et al. 1998; Malo et al. 2005). Similarly, males with greater sperm viability typically outcompete rival males during sperm competition (García-González and Simmons 2005). Our findings of increased sperm swimming speed and viability in dominant males, therefore, points toward dominant males having better quality ejaculates. Importantly, these findings are not consistent with the idea that subordinate males mate in disfavored roles, experience increased sperm competition risk, and are, therefore, under selection to increase sperm quality (Parker 1990). Contrary to expectations, neither sperm number nor sperm morphology differed between dominant and subordinate males. This is surprising as both sperm number and sperm morphology, albeit to a lesser extent, can influence male fertility (Simmons and Fitzpatrick 2012). The lack of a social-status-mediated difference in sperm numbers could be an artifact of the technique used to collect ejaculates as manually extracting sperm may not accurately reflect male use of available sperm reserved during mating. Changes in sperm morphology, on the other hand, may require social interaction that spans longer periods of time (e.g., the duration of the spermatogenic cycle; Billard 1969). Nevertheless, the observed differences in sperm swimming speed and in viability suggest that these ejaculate traits are more responsive to behavioral interactions among males, possibly mediated by the effects of the nonsperm components of the ejaculate (e.g., rapid change in seminal fluid composition; Bartlett et al. 2017). A key next step is to uncover the mechanistic explanation(s) for the social-status-mediated differences in ejaculates we uncovered in pygmy halfbeaks, as well as the further consequences on fertilization competitiveness.

The positive relationship between precopulatory and postcopulatory competitive abilities in pygmy halfbeaks could arise from at least two, nonmutually exclusive explanations. Dominant males may be in better condition and consequently be able to invest more in both traits that allow them to ascend to socially dominant positions and in ejaculate traits (i.e., social status condition dependence of ejaculate traits; Simmons and Kotiaho 2002; Schulte-Hostedde and Millar 2004; Schulte-Hostedde et al. 2005). Indeed, several metrics of male condition suggested that dominant males were in better condition than subordinate males at the end of our experimental treatments. Dominant males grew faster and were in better condition at the end of the experiment than their initially size-matched subordinate counterparts. Furthermore, dominant males had relatively larger livers. In teleost fishes, the liver is an important store for energy reserves and relative liver mass, thus, a reliable proxy for a fish’s energy status (Tyler and Dunn 1976; Wootton et al. 1978; Culbert and Gilmour 2016). Our finding of larger livers in dominant halfbeak males, therefore, suggests that males of high social status may possess larger energy reserves. Although within-dyad differences in male condition did not predict differences in male sperm swimming speeds, our finding of condition differences between dominant and subordinate males does point toward a possible, but complex, relationship between condition and ejaculate quality. Alternatively, male–male agonistic interactions may have led to increased levels of stress in subordinates, which, in turn, impaired investment in reproductive traits. Socially subordinate males often produce higher levels of stress hormones (e.g., cortisol; Ejike and Schreck 1980; Øverli et al. 1999; Culbert and Gilmour 2016; Culbert et al. 2018), which can reduce the production of sex hormones (Ely and Henry 1978; Monder et al. 1994; Ellis 1995; Tilbrook et al. 2000; Montrose et al. 2008). Such socially induced stress can reduce sperm production and ejaculate quality in subordinate males (D’Amato 1988; Tilbrook et al. 2000), although the specific enzymatic pathways are unclear in fish (Tokarz et al. 2013; Pankhurst 2016; Tsachaki et al. 2017). In our experiment, dominant and subordinate males had equal values of sperm swimming speed and viability when agonistic interactions were reduced. However, when agonistic interactions were frequent, values for sperm swimming speed and viability dropped in subordinate males in comparison to the “+ Refuge” treatment, lending some support to the idea that overall sperm quality of subordinates was reduced when fights were frequent. Lastly, our finding is in particular mirrored by a study in the cockroach *Nauphoeta cinerea*, where social experience of males, but not social status, influenced sperm viability and spermatophore size and where the stress of social interactions reduced ejaculate size and number of sperm inseminated (Montrose et al. 2008). Further work should address these possible alternative explanations in greater detail.

The presence of available females has long been recognized to shape male investment in reproductive traits, with males increasing male sex hormone production (Olsén et al. 2006; Villagrán and Ungerfeld 2013), sperm production (Olsén and Liley 1993; Bozynski and Liley 2003; also see Devigili et al. 2015), and sperm swimming speed (Gasparini et al. 2009). Yet, in our experiment, socio-sexual environment did not influence male investment in reproductive traits and had little impact on male condition traits. Contrary to expectations, sperm swimming speed and testes mass were not influenced by male access to females. This suggests that social interactions between males are the salient cue influencing male investment in reproductive traits and that mating opportunities are less important in modulating patterns of male investment in ejaculate traits. Wild Singaporean populations of pygmy halfbeaks live in social groups ranging in size from 6 to 128 members (Devigili A, Fernlund Isaksson E, personal observation). Males in these social groups are typically found in the presence of females and may, therefore, tailor their reproductive traits based on the outcome of male–male interactions rather than based on the presence, absence, and relative access to females. Alternatively, male halfbeaks may have relatively stable social roles and, therefore, do not dynamically adjust their ejaculate quality. Allocation of resources into ejaculate traits might be bound to other factors, constraining adaptive postcopulatory responses to changes in male socio-sexual environments. The only trait we measured that was influenced by access to females was frequencies of male agonistic behaviors, with males fighting more frequently in the absence of a female. Perhaps, the lack of a need to invest time into courtship displays and matings allowed males to instead allocate resources into more frequent male–male aggressions.

Our study demonstrates that changes in the opportunity for male–male behavioral interactions can have wide-ranging consequences for how males invest in reproductive traits. This finding highlights the importance of controlling, analyzing, and varying the surrounding environment when measuring relationships across complex traits. Contrary to expectations from sperm competition theory (Parker 1990), we found evidence of a positive relationship between precopulatory and postcopulatory competitiveness in the pygmy halfbeak. Instead, our findings support the hypotheses that condition dependence of trait expenditure and/or increased stress in subordinates may lead to positive correlations between social status and ejaculate quality.

## Supplementary Material

araa118_suppl_Supplementary_files

araa118_suppl_Supplementary_Image

## Notes

Data availability: Analyses reported in this article can be reproduced using the data provided by Reuland et al. (2020).
