# Corrigendum to: Male–male behavioral interactions drive social-dominance mediated differences in ejaculate traits

**DOI:** 10.1093/beheco/arab006

**Published:** 2021-03-10

**Authors:** Charel Reuland, Brett M Culbert, Erika Fernlund Isaksson, Ariel F Kahrl, Alessandro Devigili, John L Fitzpatrick

In the originally published version of this manuscript, errors in the Results section were noted and listed in this corrigendum.

Two superfluous and one erroneous reference to figures were removed in the following text: 

“Furthermore, no differences in testes mass were recorded between males of contrasting social status (Figure 1; Table 1a)” should be “Furthermore, no differences in testes mass were recorded between males of contrasting social status (Table 1a)”.

“dominant and subordinate males were detected in the “+ Refuge” treatment (Figure 2; Table 1b). Neither male social status nor presence or absence of a refuge influence sperm count or sperm morphology (sperm head, midpiece, flagellum, and total length; Figure 2; Table 1b)” should be “dominant and subordinate males were detected in the “+ Refuge” treatment (Figure 3; Table 1b). Neither male social status nor presence or absence of a refuge influence sperm count or sperm morphology (sperm head, midpiece, flagellum, and total length; Table 1b”

The axis on Figure 3B should be corrected as follows:


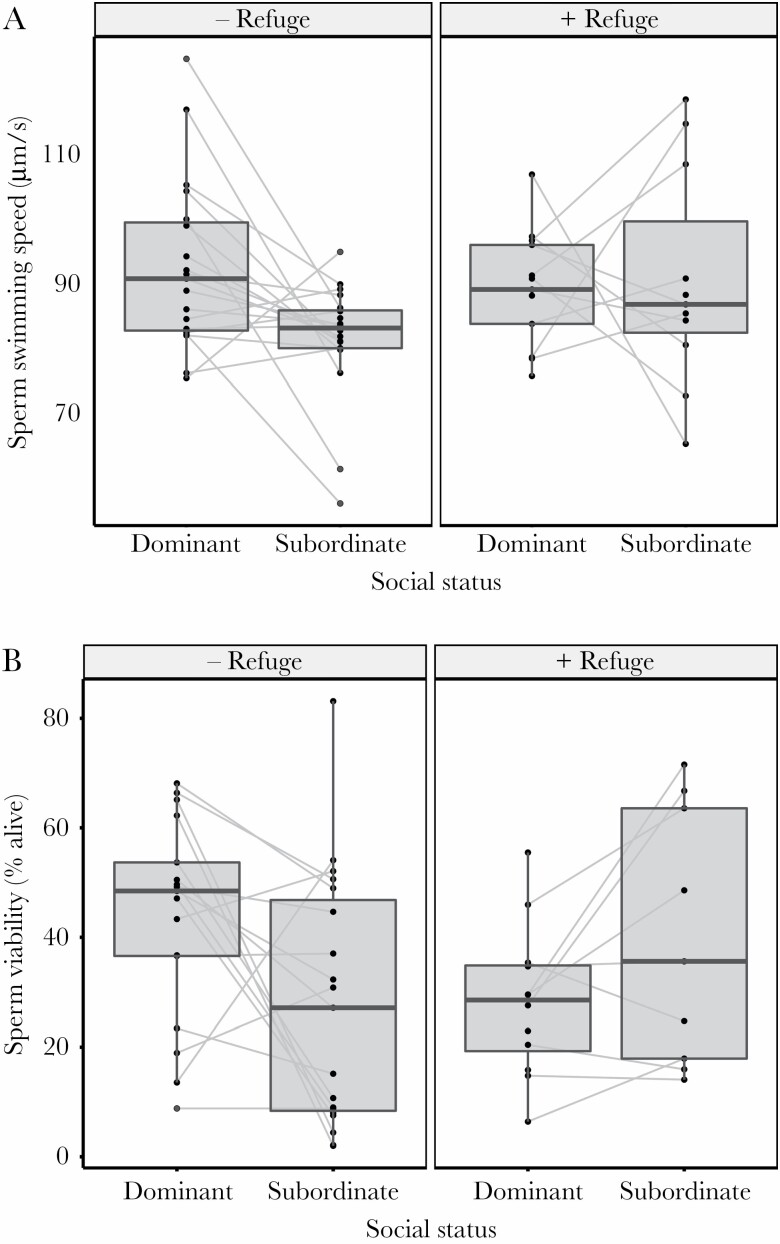


These have now been corrected online.

