# Supplementary material for: Male–male behavioral interactions drive social-dominance-mediated differences in ejaculate traits
Source: Behav Ecol. 2020 Nov 28;32(1):168–77. doi: 10.1093/beheco/araa118 (PMC7937186; doi:10.1093/beheco/araa118)
Supplement: araa118_suppl_Supplementary_files [file araa118_suppl_supplementary_files.docx]

**Experimental evidence that male-male behavioral interactions drive social-dominance mediated differences in ejaculate traits**

**Supplementary Information**

**Supplementary Table S1:** Ethogram describing behaviours performed by male pygmy halfbeaks during male-male interactions.

**Supplementary Table S2:** Experiment 2: The effect of social status and male-male interactions on key ejaculate traits – non standardized values and models with less strict dominance hierarchy cut-offs.

**Supplementary Table S3:** Experiment 2: The effect of social status and male-male interactions on key ejaculate – separate models for refuge treatments.

**Supplementary Figure S1:** Diagrams of experimental aquaria.

**Supplementary Figure S2:** Differences in male-male agonistic interaction frequencies according to ‘access to female’ treatment.

**Supplementary Figure S3:** The effect of social status and male access to females on investment in reproductive and condition traits.

**Supplementary Table S1: Ethogram describing behaviours performed by male pygmy halfbeaks during male-male interactions.** Behaviors were recorded by applying a continuous sampling method and recording all the activity that occurred while the dyads were observed. Direct agonistic interactions comprised gill flares, parallel swimming, chasing, beak-locking and biting. Recordings of males approaching and escaping were not directly incorporated into dominance indexes or male-male agonistic interaction frequencies, but are behavioral prerequisites needed to record displacements and chases.

| **Behavior** | **Description** |
| --- | --- |
| Approach | The animal swims towards a conspecific and tries to close the distance to less than a body length. |
| Escape | The animal dashes quickly away from the opponent and tries to increase the distance between the two individuals to at least more than a body length. |
| Displacement | An individual *approaches* the animal or exerts an agonistic behavior. The animal tries to increase the distance between the two animals to more than 2/3 of a body length again. Increasing of the distance has to take place within four seconds or less after an approach or an agonistic behavior. |
| Gill flare | The pelvic fins are splayed out in a v-shaped fashion. The gill covers are splayed out. Oftentimes, the beak is slightly open and the dorsal and anal fins are splayed out, making their coloration fully visible. Additionally, in a more intense version, A more intense version, the beak is fully open and the gill covers are fully splayed out. In addition, the breast fins are splayed out in a 90-degree (or more) angle. |
| Parallel swimming | Two individuals are oriented parallel to each other, either head to head and tail to tail (parallel orientation) or head to tail (antiparallel orientation). The movements of the two animals are synchronised, if one moves forward, the other mimics its movement until the two animals are in a parallel orientation again. |
| Chasing | The conspecific *escapes*. The individual follows the conspecific with comparable speed and tries to keep the distance between the individuals at least 1 ½ body length or to close the distance even further. |
| Beak-locking | The animals locks its beak around the beak of a conspecific. Either, only the lower jaw of the conspecific is held in this fashion, or both upper-and lower jaw of the conspecific are held. This position allows the animals to wrestle for an extended period of time. |
| Biting | The individual reduces the distance between it and another individual rapidly, the mouth is open. The individual appears to touch the conspecific with its beak/mouth. |

**Supplementary Table S2: Experiment 2:** **The effect of social status and male-male interactions on key ejaculate traits – non standardized values and models with less strict dominance hierarchy cut-offs.** (a) Analysis incorporating non-standardized values. (b) Analysis incorporating dyads where dominant males displaced rival males in >50 % of the interactions. In comparison to the main model where only dyads were included where dominant males won over 70 % of the fights, 8 dyads where dominant males won between 50 – 70 % of the interactions (score range 0.54 – 0.70) are reincluded. The number of experimental dyads, mean values of response variables for dominant and subordinate males, and the sample size (N) of dominant and subordinate males are presented for each model. Sample sizes (N) differ from the total number of dyads in cases where data could not be collected from males due to technical issues. Non-significant interaction terms were dropped from all final models. Significant effects are presented in bold text.

|  | **Response Variable** | **Dyads** | **Social status** | **N** | **Mean (± SE)** | **Predictors** | **χ^2^** | **p** |
| --- | --- | --- | --- | --- | --- | --- | --- | --- |
| **(a) Experiment 2: Non-standardized values** | | |  |  |  |  |  |  |
|  | Sperm swimming speed | 34 | Dominant | 32 | 91.31 ± 2.05 µm/s | Dominance status | 0.95 | 0.33 |
|  |  |  | Subordinate | 31 | 84.70 ± 2.31 µm/s | Refuge | 1.10 | 0.29 |
|  |  |  |  |  |  | Dominance status x Refuge | 10.66 | **<0.01** |
|  | Sperm viability ^a^ | 32 | Dominant | 30 | 36.03 ± 3.46 % alive | Dominance status | 0.94 | 0.33 |
|  |  |  | Subordinate | 28 | 31.69 ± 4.42 % alive | Refuge | 0.11 | 0.74 |
|  |  |  |  |  |  | Dominance status x Refuge | 4.00 | **0.046** |
|  | Sperm count | 36 | Dominant | 32 | 220262 ± 37248 cells | Dominance status | 0.50 | 0.48 |
|  |  |  | Subordinate | 33 | 187522 ± 27089 cells | Refuge | 18.44 | **<0.001** |
|  | Sperm head length | 35 | Dominant | 32 | 3.89 ± 0.02 µm | Dominance status | 0.66 | 0.42 |
|  |  |  | Subordinate | 32 | 3.87 ± 0.02 µm | Refuge | 15.58 | **<0.001** |
|  | Sperm midpiece length | 35 | Dominant | 32 | 2.31 ± 0.04 µm | Dominance status | 0.95 | 0.33 |
|  |  |  | Subordinate | 32 | 2.26 ± 0.04 µm | Refuge | 0.46 | 0.50 |
|  | Sperm tail length | 35 | Dominant | 32 | 31.66 ± 0.12 µm | Dominance status | 0.83 | 0.37 |
|  |  |  | Subordinate | 32 | 31.81 ± 0.11 µm | Refuge | 0.83 | 0.37 |
|  | Total sperm length | 35 | Dominant | 32 | 37.85 ± 0.13 µm | Dominance status | 0.31 | 0.58 |
|  |  |  | Subordinate | 32 | 37.94 ± 0.11 µm | Refuge | 1.91 | 0.17 |
| **(b) Experiment 2: All dyads** | | |  |  |  |  |  |  |
|  | Sperm swimming speed | 41 | Dominant | 39 | 91.88 ± 1.93 µm/s | Dominance status | 1.45 | 0.23 |
|  |  |  | Subordinate | 38 | 84.96 ± 2.18 µm/s | Refuge | 2.14 | 0.14 |
|  |  |  |  |  |  | Dominance status x Refuge | 5.53 | **0.019** |
|  | Sperm viability | 38 | Dominant | 36 | 36.96 ± 3.38 % alive | Dominance status | 0.51 | 0.48 |
|  |  |  | Subordinate | 34 | 35.30 ± 4.34 % alive | Refuge | 0.005 | 0.94 |
|  | Sperm count | 44 | Dominant | 40 | 217224 ± 34115 cells | Dominance status | 0.08 | 0.78 |
|  |  |  | Subordinate | 41 | 201020 ± 27827cells | Refuge | 0.85 | 0.36 |
|  | Sperm head length | 42 | Dominant | 39 | 3.88 ± 0.02 µm | Dominance status | 0.07 | 0.79 |
|  |  |  | Subordinate | 39 | 3.88 ± 0.02 µm | Refuge | 0.04 | 0.85 |
|  | Sperm midpiece length | 42 | Dominant | 39 | 2.31 ± 0.03 µm | Dominance status | 2.57 | 0.11 |
|  |  |  | Subordinate | 39 | 2.25 ± 0.03 µm | Refuge | 0.05 | 0.82 |
|  |  |  |  |  |  | Dominance status x Refuge | 4.78 | **0.029** |
|  | Sperm tail length | 42 | Dominant | 39 | 31.73 ± 0.11 µm | Dominance status | 0.17 | 0.68 |
|  |  |  | Subordinate | 39 | 31.79 ± 0.10 µm | Refuge | 0.00 | 1.00 |
|  | Total sperm length | 42 | Dominant | 39 | 37.91 ± 0.11 µm | Dominance status | 0.00 | 0.98 |
|  |  |  | Subordinate | 39 | 37.91 ± 0.11 µm | Refuge | 0.00 | 1.00 |

^a^ The interaction term for sperm viability was significant (χ^2^_(1,57)_ = 7.96, p < 0.01) when data from one outlier male with low sperm viability (0.44 %) was removed from the analysis.

**Supplementary Table S3: Experiment 2: The effect of social status and male-male interactions on key ejaculate – separate models for refuge treatments.** (a) Analysis incorporating only ‘- Refuge’ trials and (b) analysis incorporating only ‘+ Refuge’ trials. The number of experimental dyads, mean values of response variables for dominant and subordinate males, and the sample size (N) of dominant and subordinate males are presented for each model. Sample sizes (N) differ from the total number of dyads in cases where data could not be collected from males due to technical issues. Significant effects are presented in bold text.

|  | **Response Variable** | **Dyads** | **Social status** | **N** | **Mean (± SE)** | **Predictors** | **χ^2^** | **p** |
| --- | --- | --- | --- | --- | --- | --- | --- | --- |
| **(a) Experiment 2: ‘- Refuge’ trials** | | | | | | | | |
|  | Sperm swimming speed | 20 | Dominant | 19 | 92.63 ± 3.03 µm/s | Dominance status | 10.09 | **<0.01** |
|  |  |  | Subordinate | 20 | 81.49 ± 2.00 µm/s |  |  |  |
|  | Sperm viability ^a^ | 20 | Dominant | 18 | 41.24 ± 4.84 % alive | Dominance status | 2.97 | 0.08 |
|  |  |  | Subordinate | 19 | 27.82 ± 5.28 % alive |  |  |  |
|  | Sperm count | 20 | Dominant | 19 | 307970 ± 52144 cells | Dominance status | 1.24 | 0.27 |
|  |  |  | Subordinate | 20 | 230926 ± 34400 cells |  |  |  |
|  | Sperm head length | 20 | Dominant | 19 | 3.93 ± 0.02 µm | Dominance status | 0.62 | 0.43 |
|  |  |  | Subordinate | 19 | 3.91 ± 0.02 µm |  |  |  |
|  | Sperm midpiece length | 20 | Dominant | 19 | 2.27 ± 0.04 µm | Dominance status | 0.05 | 0.82 |
|  |  |  | Subordinate | 19 | 2.28 ± 0.04 µm |  |  |  |
|  | Sperm tail length | 20 | Dominant | 19 | 31.80 ± 0.12 µm | Dominance status | 0.008 | 0.93 |
|  |  |  | Subordinate | 19 | 31.79 ± 0.10 µm |  |  |  |
|  | Total sperm length | 20 | Dominant | 19 | 37.99 ± 0.13 µm | Dominance status | 0.003 | 0.95 |
|  |  |  | Subordinate | 19 | 37.98 ± 0.10 µm |  |  |  |
| **(b) Experiment 2*:* ‘+ Refuge’ trials** | | | | | | | | |
|  | Sperm swimming speed | 14 | Dominant | 13 | 89.38 ± 2.44 µm/s | Dominance status | 3.49 | *0.08* |
|  |  |  | Subordinate | 11 | 90.54 ± 5.09 µm/s |  |  |  |
|  | Sperm viability | 12 | Dominant | 12 | 28.22 ± 3.94 % alive | Dominance status | 1.98 | 0.16 |
|  |  |  | Subordinate | 9 | 39.86 ± 7.75 % alive |  |  |  |
|  | Sperm count | 16 | Dominant | 13 | 92073 ± 23563 cells | Dominance status | 0.85 | 0.36 |
|  |  |  | Subordinate | 13 | 120747 ± 38361 cells |  |  |  |
|  | Sperm head length | 15 | Dominant | 13 | 3.80 ± 0.03 µm | Dominance status | 0.06 | 0.81 |
|  |  |  | Subordinate | 13 | 3.80 ± 0.03 µm |  |  |  |
|  | Sperm midpiece length | 15 | Dominant | 13 | 2.38 ± 0.06 µm | Dominance status | 2.49 | 0.13 |
|  |  |  | Subordinate | 13 | 2.24 ± 0.07 µm |  |  |  |
|  | Sperm tail length | 15 | Dominant | 13 | 31.46 ± 0.24 µm | Dominance status | 1.35 | 0.26 |
|  |  |  | Subordinate | 13 | 31.84 ± 0.24 µm |  |  |  |
|  | Total sperm length | 15 | Dominant | 13 | 37.63 ± 0.24 µm | Dominance status | 0.51 | 0.48 |
|  |  |  | Subordinate | 13 | 37.87 ± 0.24 µm |  |  |  |

^a^ The effect of dominance status on sperm viability was significant (χ^2^_(1,36)_ = 8.42, p < 0.01) when data from one outlier male with low sperm viability (0.44 %) was removed from the analysis.


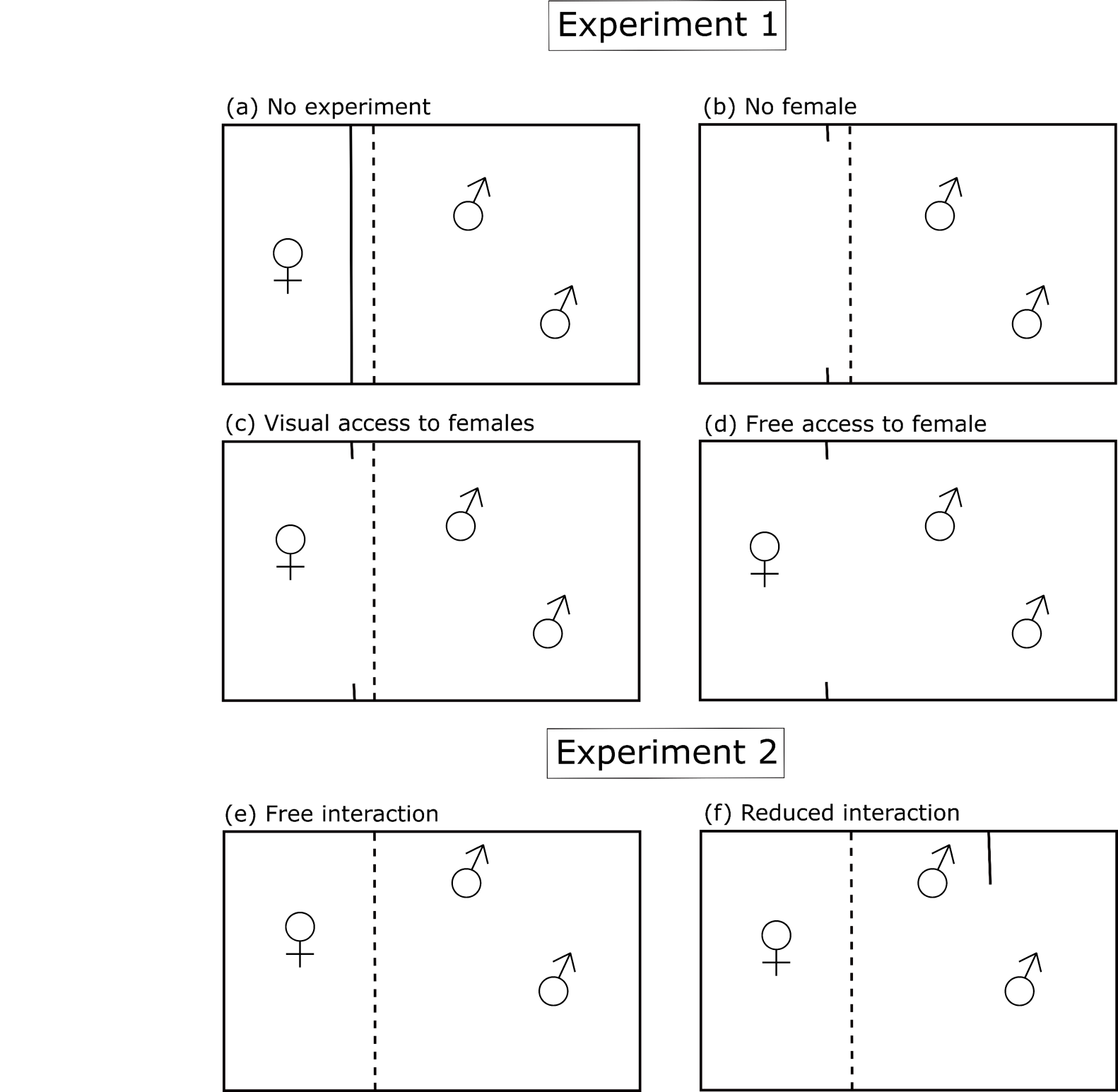


**Supplementary Figure S1: Diagrams of experimental aquaria.** Experimental aquaria (40 x 25 x 30 cm) in Experiment 1 and 2 were partitioned into two chambers, denoted by a dotted line.
Male dyads were placed in the larger chamber (26 x 25 x 30 cm, here oriented to the right) and the female in the smaller chamber (14 x 25 x 30 cm, here oriented to the left). Partitions differed among the experiments and treatments. In Experiment 1: (a) before the first observation was recorded, the female (if present) was separated from the males via both an opaque (solid line) and a transparent partition (dashed line. Not present for (d)). (b) From the first observation onwards, the opaque partition was lifted. No female was present. (c) In the ‘visual access to female’ treatment, the opaque partition was lifted but not the transparent partition, allowing for visual but not physical contact. (d) In the ‘free access to female’ treatment, the opaque transition as lifted allowing for both physical and visual interaction of both sexes. Experiment 2: females were separated from the male dyads through a transparent partition (dashed line), allowing for visual, but never physical, contact between both sexes at all times. (e) ‘- Refuge’ trials offered only limited hiding spots for males through artificial floating plants. (d) In the ‘+ Refuge’ trials, an additional refuge was created through the inclusion of a small (5 cm length, 30 cm height) opaque wall in the male chamber (solid line).


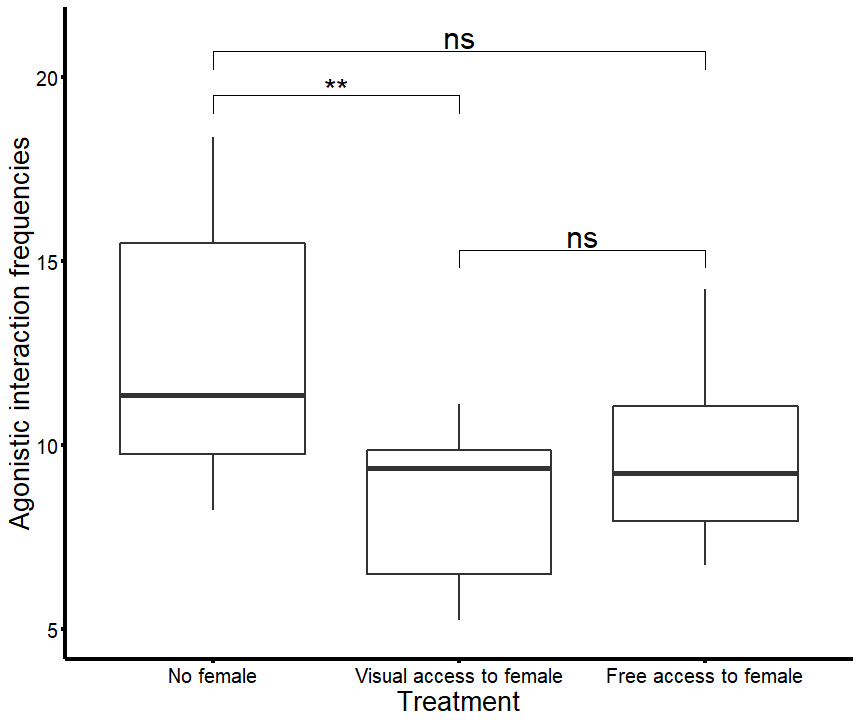


**Supplementary Figure S2: Differences in male-male agonistic interaction frequencies according to ‘access to female’ treatment.** The average frequencies of agonistic interactions per 20-minute observation is presented for males in the free access to female treatment, the no female treatment, and the visual access to female treatment. Box plots show the 10^th^, 25^th^, 50^th^ (median), 75^th^ and 90^th^ percentiles of agonistic interactions between males within the dyads in each access to female treatment. Data from one dyad with high level of agonistic interactions, about 2.2x higher than the average, was removed from the visual representation and analysis. Agonistic interactions between males were more frequently observed when males had neither visual nor physical contact with females (Tukey post hoc test: No female vs. visual access to female *t*(28) = 3.34, p < 0.01; No female vs. free access to female *t*(28) = -2.42, p = 0.057; visual access vs free access to female *t*(28) = 0.97, p = 0.60).


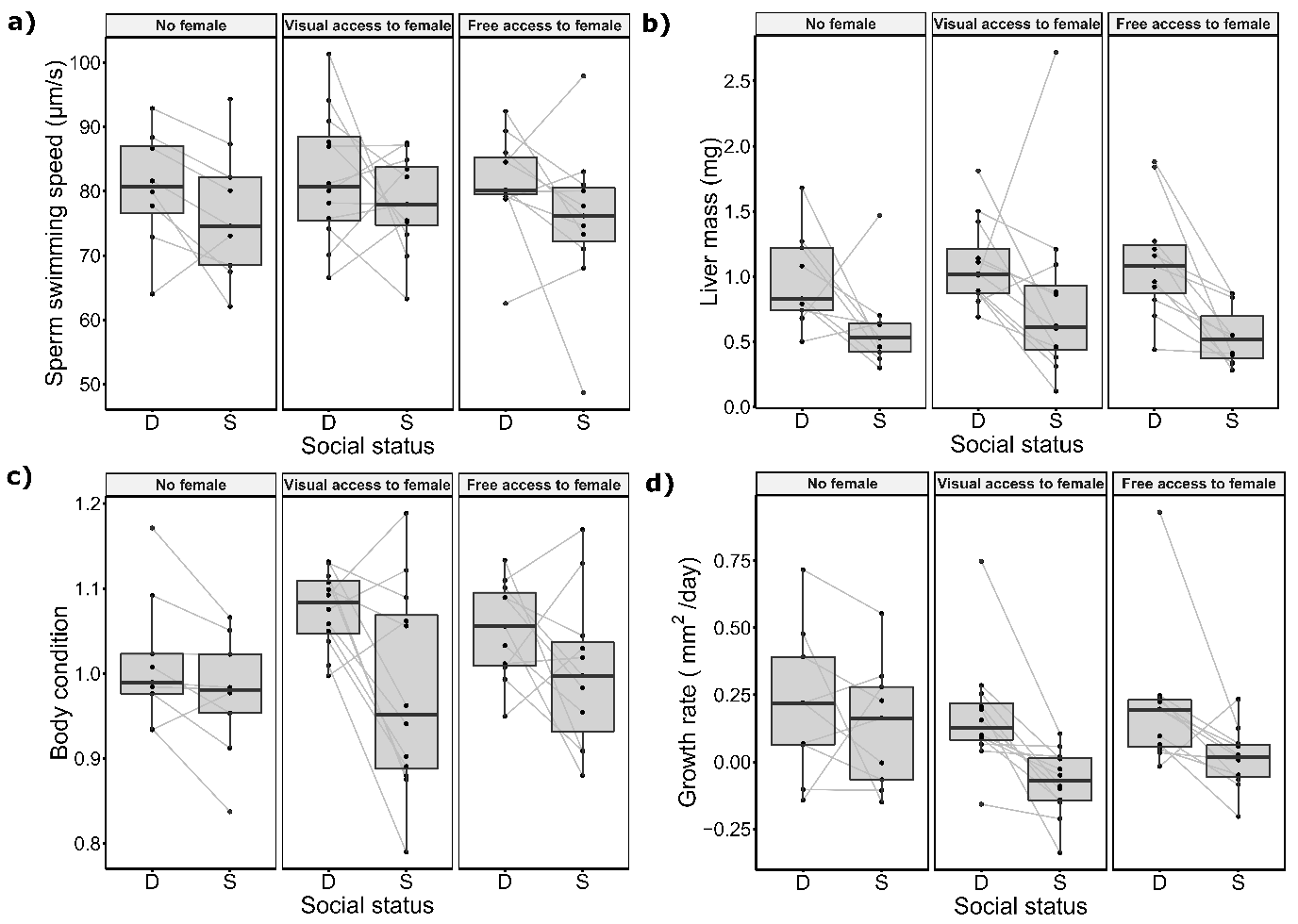


**Supplementary Figure S3: The effect of social status and male access to females on investment in reproductive and condition traits.** Individual values are presented for dominant (D) and subordinate (S) males for a) sperm swimming speed, b) liver mass, c) body condition at the end of the experiment, and d) growth rates during the experiment. Points represent individual values and males within the same dyad are linked by a grey line. Lines are absent in cases where values for only one male within a dyad were present. Box plots summarize the 10^th^, 25^th^, 50^th^ (median), 75^th^ and 90^th^ percentiles among males classified as being socially dominant (D) or subordinate (S) in each respective treatment (no female, visual access to female, and free access to female). Note that although raw liver mass is reported for size-matched dyads, statistical analyses accounted for male body mass.
