# Supplementary figures and images for: Male–male behavioral interactions drive social-dominance-mediated differences in ejaculate traits
Source: Behav Ecol. 2020 Nov 28;32(1):168–77. doi: 10.1093/beheco/araa118 (PMC7937186; doi:10.1093/beheco/araa118)

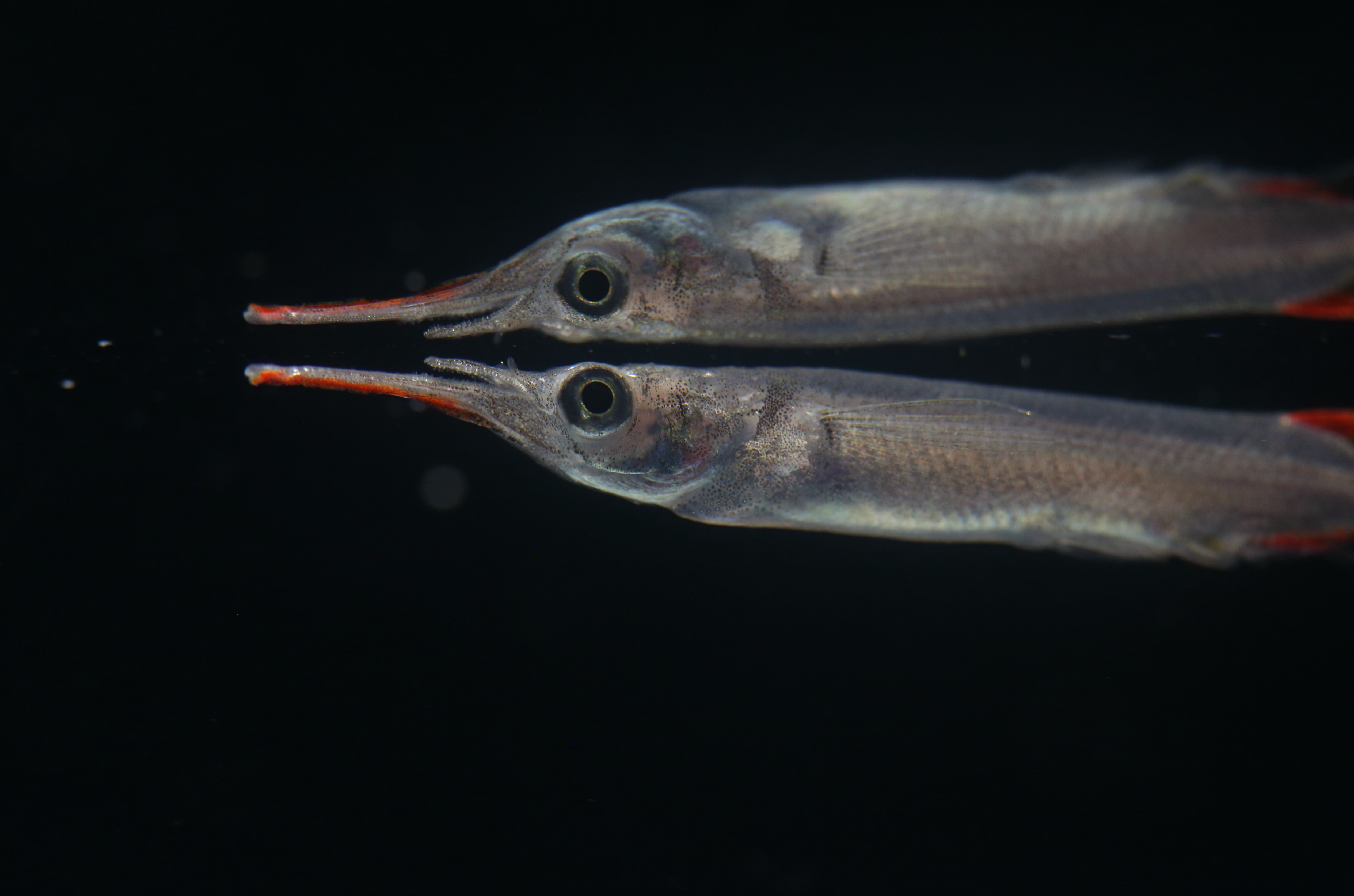

Supplement: araa118_suppl_Supplementary_Image [file araa118_suppl_supplementary_image.jpeg]
